# Expanding known viral diversity in the healthy infant gut

**DOI:** 10.1038/s41564-023-01345-7

**Published:** 2023-04-10

**Authors:** Shiraz A. Shah, Ling Deng, Jonathan Thorsen, Anders G. Pedersen, Moïra B. Dion, Josué L. Castro-Mejía, Ronalds Silins, Fie O. Romme, Romain Sausset, Leon E. Jessen, Eric Olo Ndela, Mathis Hjelmsø, Morten A. Rasmussen, Tamsin A. Redgwell, Cristina Leal Rodríguez, Gisle Vestergaard, Yichang Zhang, Bo Chawes, Klaus Bønnelykke, Søren J. Sørensen, Hans Bisgaard, Francois Enault, Jakob Stokholm, Sylvain Moineau, Marie-Agnès Petit, Dennis S. Nielsen

**Affiliations:** 1 Copenhagen Prospective Studies on Asthma in Childhood, Copenhagen University Hospital, Herlev-Gentofte, Gentofte, Denmark; 2 Department of Food Science, University of Copenhagen, Copenhagen, Denmark; 3 Novo Nordisk Foundation Center for Basic Metabolic Research, Faculty of Health and Medical Sciences, University of Copenhagen, Copenhagen, Denmark; 4 Department of Health Technology, Technical University of Denmark, Lyngby, Denmark; 5 Département de biochimie, de microbiologie, et de bio-informatique, Faculté des sciences et de génie, Université Laval, Québec City, Quebec Canada; 6 Groupe de recherche en écologie buccale, Faculté de médecine dentaire, Université Laval, Québec City, Quebec Canada; 7 Université Paris-Saclay, INRAE, Agroparistech, Micalis institute, Jouy-en-Josas, France; 8 Lab de Microorganismes: Génome et Environnement, Université Clermont Auvergne, Clermont-Ferrand, France; 9 Department of Biology, University of Copenhagen, Copenhagen, Denmark; 10 Félix d’Hérelle Reference Center for Bacterial Viruses, Université Laval, Québec City, Quebec Canada

**Keywords:** Phage biology, Translational research

## Abstract

The gut microbiome is shaped through infancy and impacts the maturation of the immune system, thus protecting against chronic disease later in life. Phages, or viruses that infect bacteria, modulate bacterial growth by lysis and lysogeny, with the latter being especially prominent in the infant gut. Viral metagenomes (viromes) are difficult to analyse because they span uncharted viral diversity, lacking marker genes and standardized detection methods. Here we systematically resolved the viral diversity in faecal viromes from 647 1-year-olds belonging to Copenhagen Prospective Studies on Asthma in Childhood 2010, an unselected Danish cohort of healthy mother–child pairs. By assembly and curation we uncovered 10,000 viral species from 248 virus family-level clades (VFCs). Most (232 VFCs) were previously unknown, belonging to the *Caudoviricetes* viral class. Hosts were determined for 79% of phage using clustered regularly interspaced short palindromic repeat spacers within bacterial metagenomes from the same children. Typical *Bacteroides*-infecting crAssphages were outnumbered by undescribed phage families infecting *Clostridiales* and *Bifidobacterium*. Phage lifestyles were conserved at the viral family level, with 33 virulent and 118 temperate phage families. Virulent phages were more abundant, while temperate ones were more prevalent and diverse. Together, the viral families found in this study expand existing phage taxonomy and provide a resource aiding future infant gut virome research.

## Abstract (web summary)

Faecal viromes of 647 healthy infants were deeply sequenced to identify more than 230 previously undescribed gut virus families, expanding known diversity within the human infant gut virome.

## Main

The establishment of the gut microbiome (GM) during the first years of life plays a pivotal role in the maturation of the infant immune system^1,2^. Early-life GM dysbiosis has been linked to a series of chronic diseases occurring later in life, indicative of a lasting effect on immune programming^3–6^. Most existing research has focused on the bacterial component of the GM, but lately it has become evident that viruses are prominent GM members. Recent studies have shown that the transfer of gut viral content from healthy donors can cure recurrent *Clostridioides* *difficile* infections^7^, alleviate diet induced obesity^8^ and prevent necrotizing enterocolitis in preterm neonates^9^. The mechanisms are still unclear, but probably involve modulation of GM composition through viral infection, because most gut viruses are bacteriophages (phages) that only infect bacteria^10^.

Phages, like bacteria, appear in the gut during the first months of life following a host-specific pattern^11–14^. Virulent phages undergo the lytic cycle in which they readily multiply and kill their host cell through lysis and release new virions into the ecosystem. Temperate phages can integrate into the bacterial chromosome, thereby becoming prophages. This prophage status postpones the killing of the host until certain environmental conditions induce the prophage to enter the lytic cycle. Some phages can also cause chronic infections leading to continuous shedding of viral particles^15^. Bacteria will defend themselves against these viruses using multiple defence systems^16^, including clustered regularly interspaced short palindromic repeat (CRISPR)–Cas systems, an adaptive immune mechanism where DNA records (spacers) of past viral infections are stored on a chromosomal CRISPR array to combat future phage attacks^17^.

Phages can alter GM composition and function^8,12^, but may also directly elicit immune responses from the human host^18–20^, suggesting a tripartite interaction that could modulate host health. The first report on the viral metagenome (virome) composition in the infant gut dates back more than a decade^21^, and the infant virome has recently been shown to be influenced by caesarean section and formula milk^22^. Nevertheless large-scale studies establishing the early life virome composition and structure are sparse, and human virome studies in general have been challenged by the large proportion of uncharted viral diversity, which is sometimes referred to as the viral ‘dark matter’ problem^23^.

The latter means that only a small fraction of nucleic acid sequences in a virome can be linked to any known virus. Attempts at de novo virus identification have been limited by the lack of universal viral marker genes, while de novo classification of novel viruses into taxa was hampered by the lack of standardized methods. However, progress has been made in recent years^24–26^, leading to several human gut virus databases^27–29^, although these are still developing and currently lack viral taxonomies for all the novel viruses they contain. Comprehensive viral taxonomies are important for conducting biologically meaningful statistical analyses against sample metadata.

Traditionally, defining new viral taxa has required laboratory isolation of both virus and host for subsequent characterization^30^. However, the International Committee for the Taxonomy of Viruses (ICTV) has recently made it possible to define new viral taxa on the basis of sequence information alone. This important change is already having major implications as several new taxa are being proposed, particularly among the highly diverse tailed phages (caudoviruses)^31^. Notably, the ICTV established the complete taxonomy of the new *Herelleviridae* family, demonstrating the definition of viral families, subfamilies and genera according to this new paradigm^32^. Subsequently, three new caudoviral families were identified in human gut metagenome data^33^. And recently, the prominent gut phage family *Crassviridae*^34^ was elevated into a viral order *Crassvirales*^35^, belonging to the new viral class *Caudoviricetes*, which itself is now proposed to encompass caudoviruses^36^ as a whole.

In this Resource, we characterized the faecal viromes of 647 infants at 1 year of age enrolled in the Copenhagen Prospective Studies on Asthma in Childhood 2010 (COPSAC2010) cohort^37^. De novo assembly and careful curation allowed us to map out any uncharted viral diversity leading to the identification of hundreds of virus family-level clades (VFCs). In contrast to the adult gut dominated by virulent *Crassvirales*, we found a diverse and largely temperate infant gut virome.

## Results

### Study population

COPSAC2010 is a population-based mother–child cohort study of 700 Danish children from rural, suburban and urban settings around the greater Copenhagen area (Supplementary Table 1). Participants were recruited in pregnancy with the aim of prospectively studying the causes for chronic inflammatory diseases^37^. Faecal samples were successfully collected and had viromes characterized for 647 children at 1 year of age. Metagenomes were sequenced in parallel^38^.

### Identifying the viruses and resolving their taxonomies

Virome extractions are known to contain various amounts of bacterial contaminating DNA^39^ and uncharted viral diversity^23^ makes it difficult to discern novel viruses from contaminants. We resolved this issue by assembly, clustering and successive rounds of manual curation (Methods), to avoid potential selection biases (for details, see Supplementary Information and Supplementary Table 2) in existing tools and criteria such as ‘circular contigs’^33^ that could have prevented the identification of truly novel viral clades.

**Extended Data Fig. 1:**
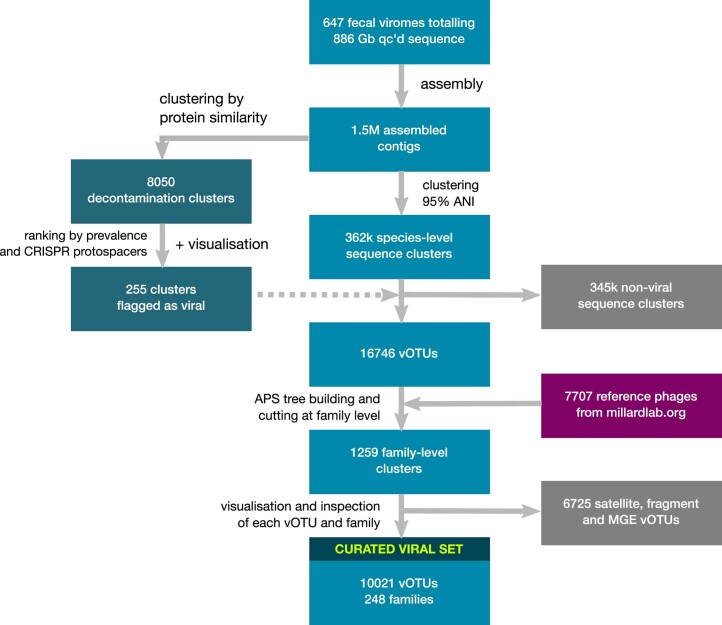
Overview of decontamination and curation procedure.

**Extended Data Fig. 2:**
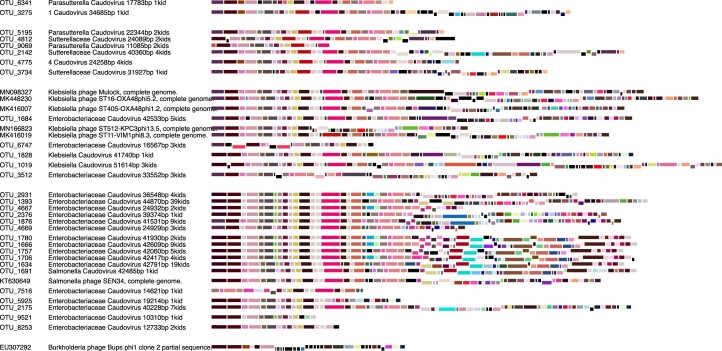
Clickable gene map of vOTUs belonging to the the *Ingridviridae* family. Available online at http://copsac.com/earlyvir/f1y/families/Ingridviridae.svg along with similar maps for the remaining 247 families, available via http://copsac.com/earlyvir/f1y/fig1.svg. Small vertical gaps between vOTUs denote genus boundaries, while large gaps denote subfamily boundaries. Ordering of the vOTUs follows the order in the APS tree and thus, related vOTUs are next to each other. ORFs are aligned vertically based on strandedness and colored by VOG affiliation. VOG definitions against the PhROGs database^87^ can be looked up by clicking on each ORF. ORF gene product (GP) numbers are displayed by mouse-over hovering. GenBank files for each vOTU can be viewed along with virus and host taxonomy by clicking on the OTU name. Caudoviral maps were inverted and zeroed according to TerL gene coordinates, while the GenBank files were not. Reference phages that belong to the same family were also included in the maps and are indicated by GenBank accession numbers.

**Extended Data Fig. 3:**
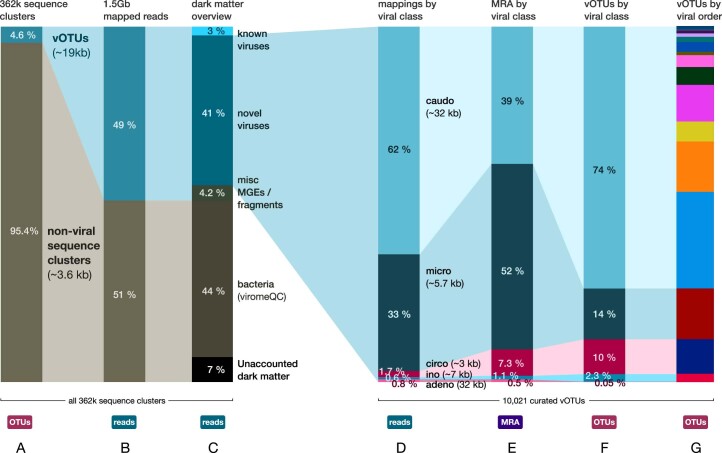
From assembly to curated vOTUs in numbers. After assembly, species-level deduplication and manual decontamination, most sequence clusters were inferred to be non-viral and had small sizes while viral OTUs were much fewer but longer (A). After mapping, vOTUs accounted for roughly half of the reads (B). 97% of the reads originally comprised “dark matter” but only 7% was left after resolution (C). The 10,021 curated vOTUs fell within five viral classes (caudoviruses [dsDNA], microviruses [ssDNA], anelloviruses [ssDNA], inoviruses [ssDNA] and adenoviruses [dsDNA]). Distributions of the viral classes by: mapped reads (D), MRAs, after normalising read counts for sequencing depth and genome size (E) and species richness, that is number of vOTUs (F) are shown. G) Same as F but at viral order-level, with orders colored as in Fig. 2.

**Extended Data Fig. 4:**
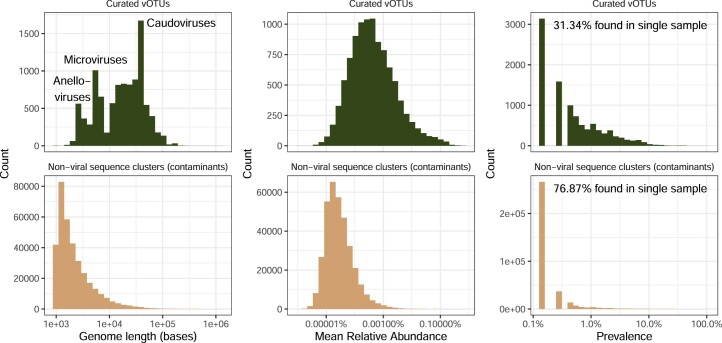
Features of vOTUs versus non-viral sequence clusters within data. Distribution of size, MRA and sample prevalence for contaminant non-viral sequence clusters and curated vOTUs respectively. The vOTU size distribution shows peaks corresponding to genome lengths for the three major classes of viruses in the dataset, namely anelloviruses, microviruses and caudoviruses (3 kb, 5.5 kb, and 40 kb). The contaminant size distribution peaks at the contig inclusion cutoff (1 kb) continuing with a long uniform tail, consistent with the unspecific origin expected for contaminating DNA. Curated vOTUs were more abundant and prevalent than contaminating species. The majority of the contaminating sequences were sample-specific, in contrast to most curated vOTUs which were found in more than one sample. The latter is consistent with their bacterial chromosomal origin, as unspecific subsampling of the large bacterial genome space is unlikely to yield overlaps between samples.

**Extended Data Fig. 5:**
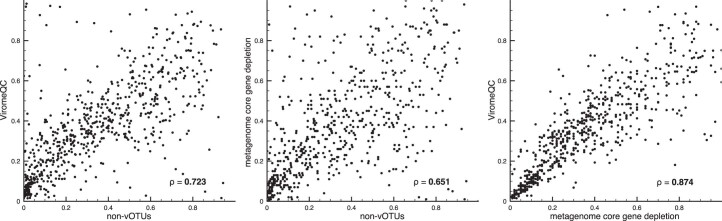
Comparison of three approaches for estimating the proportion of bacterial contamination. Each graph has 647 dots, one for each sample. Axes denote the proportion of bacterial contamination as estimated by the indicated method. Each graph is a pairwise comparison of two different methods. A) mappings to non-viral sequence clusters versus ViromeQC B) non-viral sequence cluster mappings versus metagenome core gene depletion C) metagenome core gene depletion versus ViromeQC. Spearman’s correlation coefficients (ρ) are given for all three comparisons.

In short, the extracted viromes were sequenced to an average of 3 Gbp per infant sample. After assembly and species-level de-duplication, resulting operational taxonomic units (OTUs) were clustered by protein content (Extended Data Fig. 1), visualised (Extended Data Fig. 2) and manually curated. Ultimately, 10,021 manually confirmed viral OTUs (vOTUs) comprised the study’s final set of viral species (for details, see Supplementary Information and Online Methods). These vOTUs recruited roughly half of total sequencing reads, with the remaining half mapping mainly to sequence clusters of bacterial contaminating DNA (Supplementary Information and Extended Data Figs. 3–5), which is comparable to other studies^40^. Contaminant sequence clusters were not analysed further.

For determining which vOTUs were parts of existing viral families, we pooled them with 7,705 species-level de-duplicated reference phages^41^. After gene calling, protein alignments were used for defining viral orthologue gene clusters (VOGs) de novo and for constructing an aggregate protein similarity (APS) tree. The tree was rooted and cut at levels reproducing the recent taxonomy for the *Herelleviridae*^32^ phage family, thus yielding clusters corresponding to viral families (VFCs), subfamilies and genera covering both vOTUs and reference phages. An additional order-level cutoff was based on the newly proposed caudoviral *Crassvirales* order^35^.

**Fig. 1:**
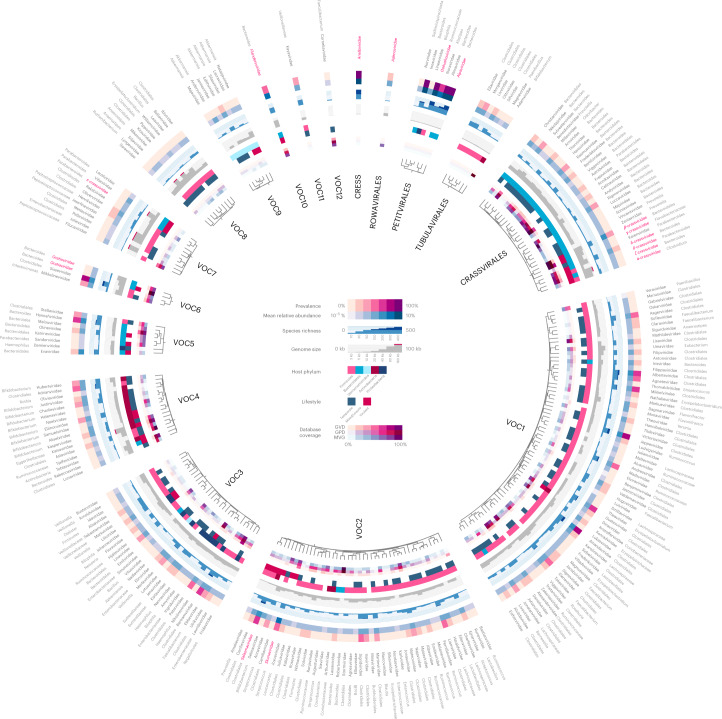
An atlas of infant gut DNA virus diversity. Faecal viromes from 647 infants at age 1 year were deeply sequenced, assembled and curated, resulting in the identification of 10,021 viral species falling within 248 VFCs. Predicted host ranges for each VFC are given, and the VFCs have been grouped into 17 VOCs. Trees show how VFCs are interrelated within each VOC, and heat maps and histograms encode their genome size, lifestyle, host range, abundance and prevalence across the cohort as well as in published gut virus databases. For the 16 previously known viral families, names are written in red. An interactive version of the figure with expandable families can be accessed online, for browsing the gene contents and downloading the genome of each virus: http://copsac.com/earlyvir/f1y/fig1.svg.

The 10,021 species-level vOTUs fell within 248 curated VFCs, including 16 known families (Fig. 1) containing 2,497 vOTUs and 232 previously undescribed VFCs containing 7524 vOTUs. The undescribed VFCs were named after the infants that delivered the faecal samples. The VFCs were additionally grouped into 17 virus order-level clusters (VOCs, Supplementary Table 3), 5 of which were already known (Fig. 1). After estimating the typical complete genome size at the family level (Fig. 1), 56 % of the 10,021 vOTUs were found to be complete or near complete, specifically, 83% of the 2,629 small single-stranded DNA (ssDNA) vOTUs and 46% of the 7,392 larger double-stranded DNA (dsDNA) vOTUs. vOTU DNA sequences and taxonomies along with visualizations of the VFCs (Extended Data Fig. 2) have been made available via an interactive Fig. 1 at http://copsac.com/earlyvir/f1y/fig1.svg.

### Infant gut vOTUs are largely absent from gut virus databases

We checked whether any of our 10,021 curated viral species were found within three gut virus databases built mainly on adult faecal metagenomic data. The Gut Virome Database (GVD)^29^ contained only 819 of our vOTUs, while the larger and more recent Gut Phage Database (GPD)^28^ and Metagenomic Gut Virus catalogue (MGV)^27^ covered 2,307 and 2,171 vOTUs, respectively. Combined, 7,046 (70%) of the infant gut vOTUs identified here were not found in any of the three gut virus databases. At the family level, however, most of the 248 VFCs had some representatives in either database, with *Crassvirales* VFCs being particularly well represented in both GPD and MGV. Importantly, the majority of our most species-rich VFCs (for example, candidate family ‘Amandaviridae’) were poorly represented in all three databases, while the VFCs best covered by the databases were often minor in our data (Fig. 1). In other words, most large gut phage clades in databases are only occasionally found in the infant gut viromes, and vice versa. This pattern suggests that the infant gut is a unique niche harbouring specialized viruses distinct from the adult gut. Alternative explanations for this lack of overlap could be library selection differences (bonafide viromics in our case versus bulk metagenomics), bioinformatics (curation versus automated detection), limited infant gut sequence diversity (enabling complete assembly of otherwise rare phages) or the fact that gut viromes are extremely individual specific by nature.

### Undescribed viral families dominate the infant gut virome

Cutting the APS tree at the family^32^ and order level^35^ yielded 248 VFCs and 17 VOCs. The family-level cutoff reproduced the recently defined crAssphage families^35^ (Fig. 1). The order-level cutoff reproduced five known viral orders (that is, *Petitvirales*, *Tubulavirales*, anelloviruses (CRESS), *Rowavirales* and *Crassvirales*) along with 12 additional strictly caudoviral VOCs (Supplementary Table 3). Even at the family level, 232 out of 248 VFCs were caudoviral, further underlining their diversity. The mean and median VFC size was 40 and 17 species-level vOTUs, respectively, making the typical VFC similar in richness to currently known gut phage families such as *Flandersviridae*^33^.

**Fig. 2:**
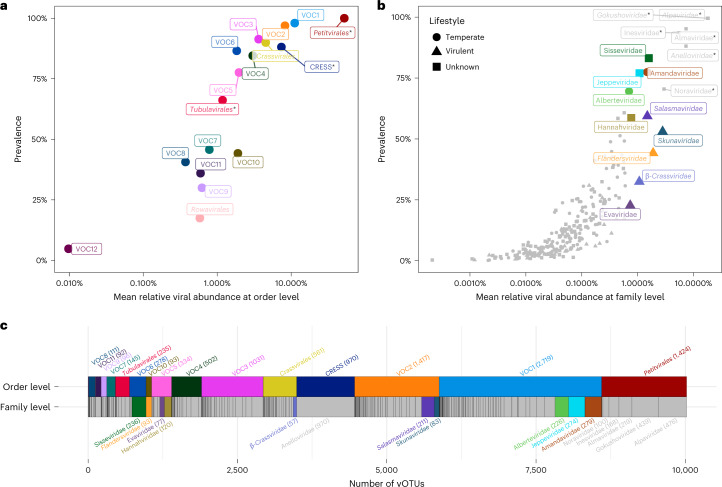
Abundance, prevalence and richness of the viral clades in the 1-year-old infant gut. Already-known viral clades are indicated in italics. ssDNA clades have been marked with a star as their abundances may be inflated from amplification bias. **a**, Prevalence and MRA of the 17 VOCs across samples. **b**, Prevalence and MRA of the 248 VFCs. The major VFCs were defined as the ten most abundant caudoviral VFCs in the data, and are coloured and labelled. Minor VFCs as well as ssDNA families are in grey. Predicted lifestyles for the ten major VFCs are indicated by different shapes. **c**, VOCs and VFCs scaled by species richness, ordered by MRA. VOC12 and Rowavirales are not shown due to their small sizes. The VFCs are represented underneath the VOCs they belong to. Clade prevalence, abundance and species richness are highly interrelated, and several previously undescribed clades outnumber crAssphage in the infant gut.

**Extended Data Fig. 6:**
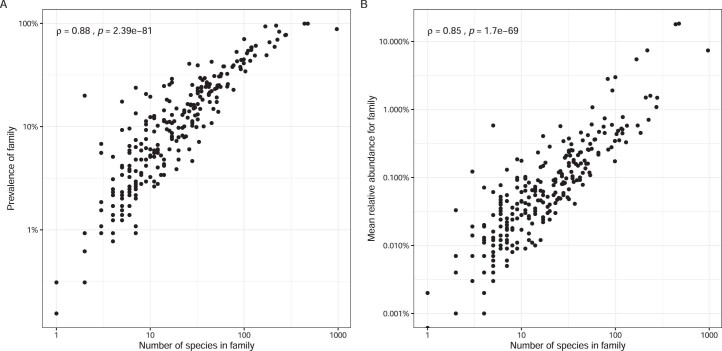
Viral family species-richness is linked to prevalence and abundance. The species-richness within a family is highly correlated with both its prevalence (A) and the MRA across samples (B), shown here with Spearman’s correlation tests (two-sided *P* values). MRAs are correspondingly correlated with prevalence as already shown in Fig. 3. The correlation between all three measures is in line with predictions made by the neutral community model. MRA, mean relative abundance.

To identify the most predominant viral clades, three measures were calculated: total species richness, prevalence across samples and mean relative abundance (MRA) (Fig. 2). Family- and order-level MRA and prevalence estimates were determined by first mapping sample reads to vOTUs, then aggregating their counts on the basis of taxonomic affiliation. All three measures were highly correlated (Fig. 2 and Extended Data Fig. 6), meaning that the most diverse VFCs and VOCs were also the most widespread and abundant. The correlation between these measures is predicted by the neutral community model, which also applies to bacterial community structures^42,43^.

In our data, vertebrate-infecting ssDNA anelloviruses (*Anelloviridae*) and bacterial ssDNA microviruses (*Petitvirales*) were amongst the most abundant viral clades (Fig. 2a and next subsection). These were followed by ten major dsDNA VFCs belonging to the *Caudoviricetes* viral class (Fig. 2b). Four of these are known caudoviral families pending ICTV approval, namely *Skunaviridae*, *Salasmaviridae*, *β-crassviridae* and *Flandersviridae*, while the remaining six comprise new candidate families. Importantly, *Crassvirales*, which are abundant in adult faecal viromes^44^, were surpassed by other VOCs in the infant gut (Fig. 2a).

*Skunaviridae* is a family of virulent phages infecting *Lactococcus* dairy cultures^45^. Possibly originating from the diet, they were the most abundant caudoviral family in our data (2.7% MRA). *Salasmaviridae* is a viral family harbouring around a dozen *Bacillus* podoviral species including phage phi29^46^. Here, we were able to broaden the scope of the *Salasmaviridae* family with over 200 diverse vOTUs spanning more than 20 viral subfamilies, infecting a wide variety of gut-associated *Firmicutes* and *Actinobacteria*. *β-Crassviridae*, a minor *Crassvirales* family in adults, was found in almost a third of the infants (*n* = 210; 647), predicted to infect both *Bacteroides* and *Clostridiales* hosts. The major adult *Crassvirales* family, *α-Crassviridae*^35,47^, however, was present in only 5% (*n* = 39) of the infants. *Flandersviridae* is a *Bacteroides*-infecting phage family recently defined on the basis of 30 complete phage genomes^33^ from public metagenome assemblies. Found in almost half of the children (*n* = 286), we markedly expand this family with 80 complete species-level vOTUs spanning four subfamilies.

Apart from these four known virulent viral families, six previously undescribed candidate families were found to be highly abundant, prevalent and diverse. The prevalence and richness estimates for these candidate families indicate that they are at least as predominant in the infant gut ecosystem as crAssphage is in adults^44^. Candidate family ‘Sisseviridae’, almost universally present in the infants (80%), harbours the highly prevalent *Faecalibacterium* phage Oengus^48^ and encompasses a wide range of both temperate and virulent vOTUs infecting diverse *Firmicutes* and *Actinobacteria*. The temperate candidate families ‘Amandaviridae’, ‘Jeppeviridae’ and ‘Alberteviridae’ are related, belonging to the major VOC1. These candidate families were present in 70% of the infants, containing between 200 and 300 viral species each, infecting *Clostridiales* genera such as *Ruminococcus*, *Blautia*, *Anaerostipes* and *Hungatella*. Apart from a few unclassified *Clostridium* and *Brevibacillus* reference phage species that co-cluster within them, these expansive clades are largely unexplored. Finally, ‘Evaviridae’ and ‘Hannahviridae’ comprise two related candidate families of *Bacteroides*-infecting phages containing around 200 species in total. The former appears strictly virulent while the latter harbours separate subfamilies that are either virulent or temperate. ‘Hannahviridae’ includes the recently described *Bacteroides* phage ‘Hankyphage’^49^ known for its diversity-generating retroelements, and it has been extensively described in a parallel provirome study performed on the same samples^50^.

### Clades of ssDNA viruses in the infant gut

**Fig. 3:**
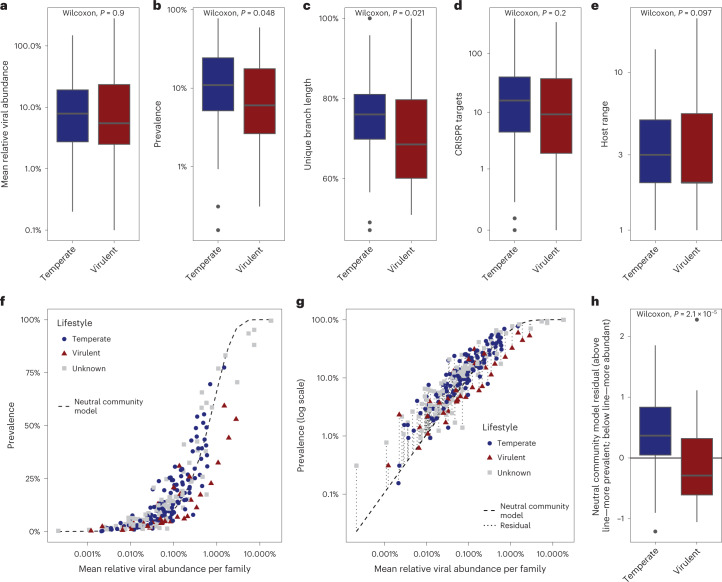
Temperate versus virulent viral families in the infant gut. **a**–**e**, Characteristics of temperate versus virulent VFCs in the data in terms of MRA (**a**), prevalence (**b**), genetic diversity as measured by unique branch length (**c**), number of metagenomic CRISPR spacer matches (**d**) and host range (number of host species) (**e**). **f**, Fit of the neutral community model, on the VFCs from Fig. 2b. **g**, Deriving neutral community model residuals from the log-transformed prevalences. **h**, Comparison of neutral community model residuals, showing that temperate VFCs tend to have positive residuals, whereas virulent VFCs tend towards negative residuals, indicating that temperate phages are present in lower abundance despite being found in more children, as compared with virulent phages. For **a**–**e** and **h**, *n* = 151 (118 temperate + 33 virulent). Box plot elements: centre line, median; box limits, upper and lower quartiles; whiskers, 1.5× IQR; points, outliers. Two-sided Wilcoxon test *P* values reported. For **f** and **g**, *n* = 248 (118 temperate + 33 virulent + 97 unknown).

ssDNA vOTUs recruited around a third of the sequencing reads, but after normalizing for their short genome sizes, they accounted for 60% of the MRA (Extended Data Fig. 3). The short multiple-displacement amplification (sMDA) protocol used to detect the ssDNA viruses could have inflated their counts^51^. However, the families did still display canonical positioning along the neutral community model (Figs. 2b and 3f) so we infer that any artificial inflation would have been limited. The ssDNA families fell within three separate viral classes, *Malgrandeviricetes*, CRESS viruses and *Faserviricetes*, each harbouring a single viral order.

Microviruses of the *Petitvirales* viral order (class *Malgrandeviricetes*) are ubiquitous small icosahedral ssDNA phages and were the most prevalent and abundant group of viruses in our viromes, making up 52% of the MRA. Twenty-one per cent of all CRISPR spacer matches from the metagenome targeted microviruses (http://copsac.com/earlyvir/f1y/taxtable.html), underlining their importance. vOTUs from the two major families *Gokushoviridae* and *Alpaviridae* (currently subfamilies *Gokushovirinae* and *Alpavirinae*) in our data are predicted to infect *Clostridiales* and *Bacteroidales,* respectively, but other minor VFCs were also detected (Fig. 1).

Anelloviruses from the CRESS class of ssDNA viruses, also known as Torque Teno viruses, comprise a single family (*Anelloviridae*) of small 3 kb ssDNA viruses that infect vertebrate cells. They cause chronic asymptomatic infections in healthy humans, with elevated titre in immunocompromised patients^52^. The immature infant immune response may explain their abundance in our samples (7% of the MRA). They comprise by far the richest single family with 970 species-level vOTUs. On average, each infant harboured ten species of *Anelloviridae*, consistent with earlier findings^13^. Unsurprisingly, no CRISPR spacer matched any *Anelloviridae* vOTUs.

Inoviruses from the *Tubulavirales* order (class *Faserviricetes*) are a ubiquitous and diverse group of filamentous phages with small ssDNA genomes^53^. Some integrate into their host genomes using integrases while others cause chronic non-lethal infections that result in the continuous shedding of viral particles^15^. Although they were diverse in our data, distributed among seven families like the *Petitvirales*, their species richness was lower at 235 vOTUs, and abundances were correspondingly lower totalling 1% MRA. Most of the inoviral families found were predicted to infect *Clostridiales*, although members of the VFC ‘Adamviridae’, appear to specifically infect *Bifidobacterium* (Fig. 1).

### Virus lifestyle determines both abundance and prevalence

Most of the ten major caudoviral VFCs lacked integrases, otherwise commonly found in less abundant VFCs. Since an integrase is an indicator of a temperate lifestyle, we investigated whether a virulent lifestyle was linked to higher abundances. First, the typical complete genome size per VFC was determined for 228 VFCs by examining the size distribution of their constituent vOTUs. The median (interquartile range (IQR)) complete genome size for the VFCs was 35 kb (30–50 kb). Using the determined minimum complete size limit per viral family (Fig. 1), 5,608 vOTUs with complete and near-complete genomes were screened for integrases (Methods). Phage lifestyles were mostly homogeneous at the family level and a total of 118 VFCs were deemed temperate, while only 33 were found to be virulent. The remaining 97 VFCs exhibited either a mixed lifestyle pattern or were uncertain due to an insufficient number of complete genomes.

Family-level abundance was not significantly linked to phage lifestyle (two-sided Wilcoxon test, *P* = 0.90; Fig. 3a), but temperate VFCs were significantly more prevalent than virulent VFCs (*P* = 0.048; Fig. 3b). Temperate phages have been shown to be more genetically diverse than their virulent counterparts^54^, so we compared the amount of unique branch length (as a fraction of total branch length) in virulent versus temperate family-level APS subtrees. Indeed, temperate caudoviral VFCs were more genetically diverse (*P* = 0.021; Fig. 3c) than virulent VFCs. *Clostridiales* hosts were particularly enriched in temperate VFCs, whereas most virulent VFCs were predicted to infect *Bacteroidales* (Fig. 1). According to our CRISPR spacer mappings, and in line with other studies^28,55^, some vOTUs appeared to infect multiple host species, genera or even families of bacteria. We checked whether spacers targeted virulent phages more often than temperate phages, or whether a virulent lifestyle was associated with a broader host range. This was not the case as both temperate and virulent families exhibited similar mean host ranges (*P* = 0.2; Fig. 3d) and numbers of targeting spacers (*P* = 0.097; Fig. 3e).

Finally, plotting the abundance and prevalence of the virulent and temperate VFCs against each other (Fig. 3f) suggested that virulent VFCs had elevated titre despite being found in fewer children. We tested this hypothesis systematically using the neutral community model (Fig. 3g), which describes the community relationship between abundance and prevalence^56^. After fitting the model on all of our VFC abundances, virulent VFCs had significantly lower residuals against it than temperate VFCs (two-sided Wilcoxon test, *P* = 2.1 × 10^−5^; Fig. 3h), confirming that they were both less prevalent and more abundant than temperate VFCs.

### Phage–host abundances are linked in spite of virus lifestyle

**Fig. 4:**
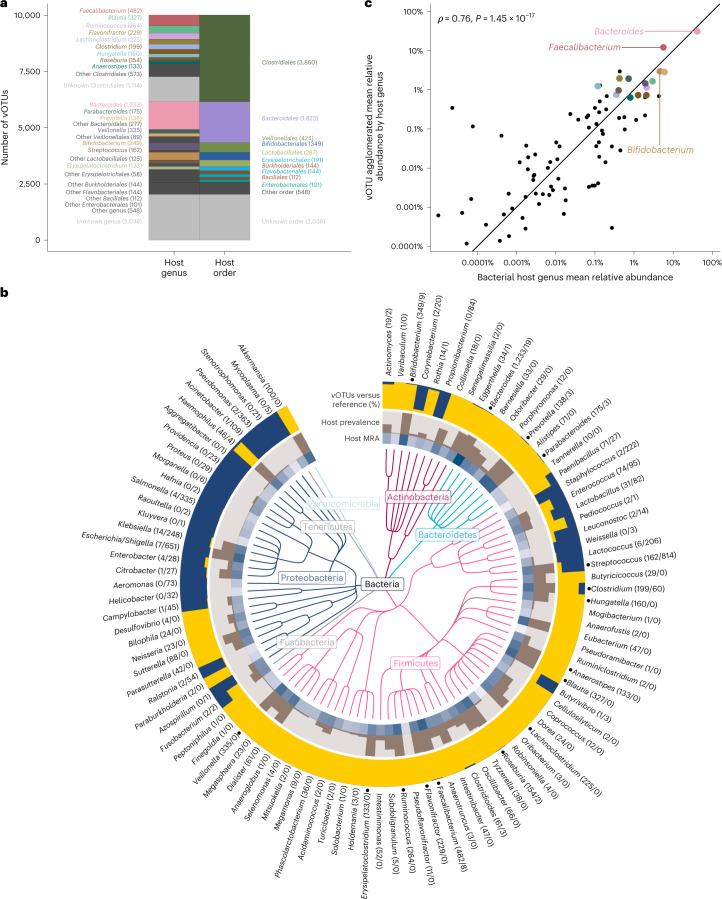
Phages and their bacterial hosts in the 1-year-old infant gut. Prediction of bacterial hosts for the 10,021 vOTUs found in the infant gut virome shows that ***Bacteroides, Faecalibacterium and Bifidobacterium*** are the three most prominent host genera. **a**, Distribution of virus host predictions collapsed to bacterial order and genus levels, respectively. Numbers in parentheses denote the number of vOTUs with a given host genus or order, respectively. **b**, The top 100 gut bacterial genera found in gut metagenomes from the same infant faecal samples, as represented by a taxonomic tree. The MRA of each bacterial genus is shown in the blue heat map, while the fraction of the 647 infants harbouring the host genus (that is its prevalence) is shown with the brown bar plot. The outer ring displays per bacterial genus, the proportion of infant gut vOTUs (yellow) relative to reference phage species with known hosts^41^ (dark blue). Numbers behind each genus name denote the total number of vOTUs versus reference phage species per bacterial host genus. The 16 major host genera from **a** are indicated by a dot in front of their names in **b**. **c**, Each dot represents a genus from **b**, by its MRA in the metagenome against the aggregate MRA of all its vOTUs in the virome. Host abundances correlated strongly with corresponding phage abundances as tested by a Spearman’s rank test (two-sided *P* value).

Bacterial hosts for the vOTUs were predicted using 317,968 CRISPR spacers from our metagenome assembled genomes (MAGs)^38^, 11 million spacers from the CRISPR spacer database^57^ and using WIsH^58^. These predictions were merged by their last common ancestor. Bacterial host genera were predicted for 63% of the vOTUs, with 77% being covered at the order level (Fig. 4a), and 79% at the host phylum level. *Bacteroides* was by far the most commonly predicted host genus followed by *Faecalibacterium* and *Bifidobacterium*. At the order level, however, approximately half of the annotated vOTUs had *Clostridiales* as hosts, with *Bacteroidales* covering just one-quarter (Fig. 4a). This mirrors the corresponding pattern for the bacterial taxa in the metagenomes, where *Bacteroides* was the most abundant genus, while *Clostridiales* were more diverse (Fig. 4b).

**Extended Data Fig. 7:**
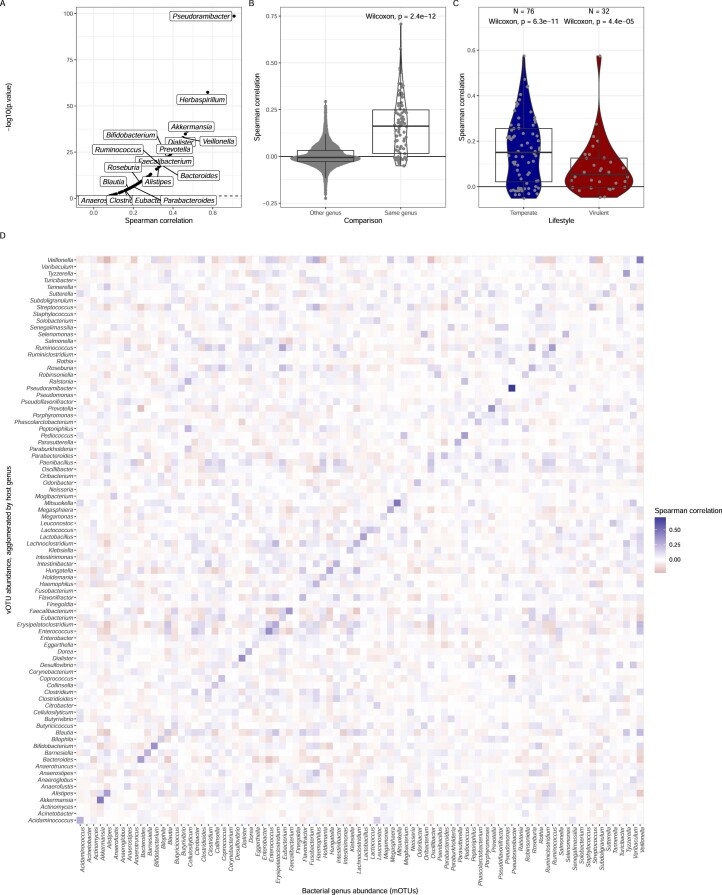
Sample-to-sample co-abundance of phages in the virome and host-bacteria in the metagenome. Correlations between host bacterial relative genus abundances in the metagenomes with aggregate relative abundances for phages predicted to infect those host genera in the virome, compared across all children. A) Volcano plot showing how all significant correlations between phage-host pairs were positive (ρ > 0; *n* = 87 genera, Spearman’s correlation tests, two-sided *P* values). B) The distribution of these correlation values was significantly higher than zero (One-sample Wilcoxon test, two-sided *P* = 2.4·10^-12^, *n* = 87, right side), whereas random non-matched phage-host pairs were centered around zero (left side). C) These correlations were positive regardless of phage lifestyle (one-sample Wilcoxon tests with two-sided *P* values), and D) stood out against the background of all genus combinations tested (same data shown in panel B, diagonal is matched phage-host pairs and off-diagonal are non-matched pairs). Boxplots demonstrate median, middle line; lower and upper quartile, box bounds; and most extreme observations within 1.5 x interquartile range above/below box, whiskers. All individual data points are overlaid on the boxplots.

**Extended Data Fig. 8:**
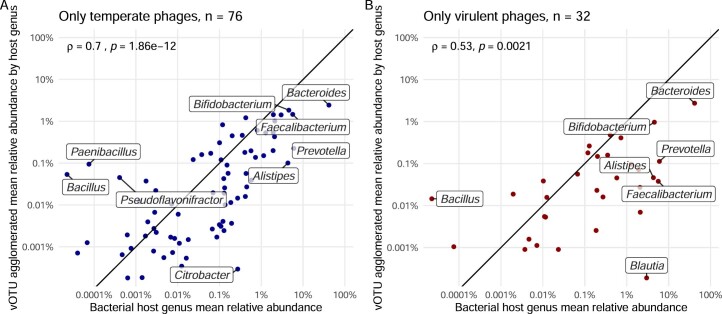
Mean co-abundance of phages and hosts regardless of viral lifestyle. Correspondence between host genus abundances in the metagenome with aggregate abundances for all phages infecting those genera in the virome, as stratified by virus lifestyle, namely, temperate phages (A) and virulent phages (B). The MRA of both virulent and temperate phages correlates positively with host MRA. MRA, mean relative abundance. Correlations were tested using Spearman’s rank test (two-sided *P* values).

MRAs of bacterial host genera in the metagenomes were strongly correlated with cognate phage MRAs in the viromes (Spearman’s *ρ* = 0.76, *P* < 1.45 × 10^−17^; Fig. 4c) supporting both the accuracies of host predictions and viral abundance estimations. Overall, in the infant gut, both virulent and temperate phages correlate positively with the abundances of their hosts (Extended Data Figs. 7 and 8). Although virulent phages lyse their hosts, cross-sectionally they still act as positive markers for their presence.

## Discussion

The recent publication of several large and curated gut virus databases illustrates the massive diversity of the human gut viral community^27–29^. Yet, significant parts of this ecological niche remain uncharacterized. A thorough description of the gut viruses is essential to understand their roles, especially if one aims at modulating the GM for prevention and treatment of chronic disease. We deeply sequenced 647 infant gut viromes and mapped the uncharted viral diversity by de novo assembly and classification. The approach led to the uncovering of 248 VFCs, 232 of which were previously unknown, and most of which belonged to the *Caudoviricetes* viral class. Temperate phages dominate the 1-year-old gut virome, and crAssphage is overshadowed by several previously undescribed viral clades. Such comprehensive taxonomic resolution of virome data allows for biologically meaningful statistical analyses against sample metadata, aiding future research in translational viromics.

Systematically resolving the uncharted viral diversity (‘dark matter’) left only 7% of the virome sequences unaccounted (Extended Data Fig. 3) and the VFCs that were uncovered in the process represent a major expansion of current phage taxonomy. Resolution of phage lifestyles showed that most phages in the infant gut ecosystem are temperate, even though the less diverse virulent phages can be more abundant. This echoes recent findings on the neonatal gut^12^, also dominated by temperate phages, and it is in contrast to adults where virulent phages dominate^29^.

In addition to the six major candidate families described, numerous additional predominant caudoviral VFCs can be browsed online (Fig. 1). In general, *Bacteroides*-infecting VFCs were more often virulent and host specific, while VFCs infecting *Clostridiales* featured wider host ranges and were overwhelmingly temperate. Multiple VFCs were often specialized for a single host genus, for example, seven *Akkermansia*-specific VFCs (Fig. 1). Others were more agnostic, having multiple host genera, for example, *Clostidiales*-infecting VFCs such as ‘Amandaviridae’. Some vOTUs were even predicted to infect multiple bacterial families within the same order. Such features underscore the rapid rates of speciation that caudoviruses attain both horizontally across hosts, but also vertically within tight host niches. Phylogenetically distinct hosts such as *Bacteroides* and *Akkermansia*^59^ probably present greater barriers for host switching, making their phage families more host specific in a human gut context. This is in contrast to *Clostridiales* genera, dozens of which often co-exist, encouraging host flexibility. Overall, we found caudoviral richness to exceed host richness by an order of magnitude, both at the species and genus levels (for example, 2,858 caudoviral genera versus 203 host genera in the metagenome).

**Extended Data Fig. 9:**
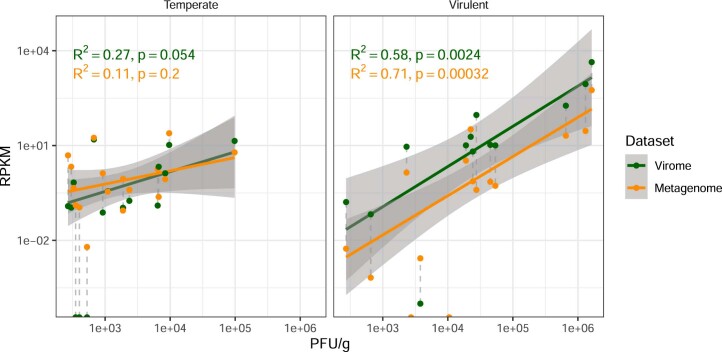
The sMDA amplified viromes are quantitative for dsDNA phages. The relationship between experimentally determined PFU/g of faeces for 32 coliphages^65^, against mapped virome and metagenome reads per kilobase per million (RPKM), from the corresponding 32 samples. The two panels show data for temperate and virulent coliphages respectively. Axes were log-transformed to capture the dynamic range. A linear model was fit following log-transformation. Temperate coliphages show only a tendency of being associated presumably because read-mappings were shared between induced phage DNA and bacterial chromosomal DNA. For the virulent coliphages, however, the relationship was quantitative throughout the range of PFU counts (from 270 to 1.6 M). The sMDA amplified virome is no less quantitative than the unamplified metagenomes for the same samples. sMDA: short multiple-displacement amplification. Paired viromes/metagenomes from the same samples are connected using dashed lines. Regression lines are drawn using linear models, the shaded area represents the 95% confidence band for the regression line. *P* values correspond to Spearman’s rank correlation tests, are two-sided and were not adjusted for multiple comparisons.

Most virome studies so far amplified the extracted DNA with MDA before sequencing, which may bias sequence composition towards ssDNA viruses^60,61^ in addition to compromising quantitative analyses overall. However, the largest meta-analysis of virome studies^29^ did not find differences between non-MDA and standard 2 h MDA gut viromes. Furthermore, in a recent gut virome study using a different DNA library kit enabling ssDNA detection supposedly without biases^61^, microviruses outnumbered caudoviruses in a third of the samples^62^. Here, we used a 30 min sMDA step to enable ssDNA detection while limiting biases. We found the opposite trend; that microviruses outnumbered caudoviruses in two-thirds of the infants. But we also showed strong co-abundances between phages and their hosts. Moreover, we made a thorough comparison linking plaque forming units to virome abundances (Extended Data Fig. 9). We conclude that our results on viral abundances are relevant in quantitative terms despite using sMDA, at least for dsDNA viruses.

*Skunaviridae*, our most abundant caudoviral family, comprised only eight complete vOTUs in the dataset. This is atypical considering the hundreds of vOTUs in most of the other abundant viral families. All reference phages belonging to the family infect *Lactococcus* while our vOTUs were predicted to infect *Streptococcus*, but this could be an artefact caused by the lack of CRISPR-Cas systems in *Lactococcus*^63^. *Streptococcus*, although very prevalent in the children, may not have been abundant enough to support the high counts of virulent *Skunaviridae*. We also did not find any strong correlation between *Skunaviridae* and *Streptococcus* or *Lactococcus* in the data. Thus, it remains a possibility that these strictly virulent phages were ingested via fermented dairy products where they naturally occur, as previously proposed^64^.

In a previous study on *Escherichia* *coli* phages isolated from the same samples^65^, virulent coliphages were less prevalent but more abundant and had broader host ranges than temperate coliphages. Here we found the same pattern on a more global scale. Virulent phage families across diverse hosts were more abundant but less prevalent than temperate phage families. Although we found no difference in host ranges, temperate phage families were more genetically diverse compared with the virulent ones. The higher prevalence and lower abundance of temperate phages probably reflects frequent prophage induction, as shown in mouse models^66–68^, and that induced virions do not readily re-infect and multiply. In viromics, this would appear as a stable background of diverse temperate phages on top of which virulent blooms would stochastically appear from random phage–host encounters. For our infant samples, this temperate background was intense enough to overshadow the diversity of virulent phages. Possibly, in adult viromes where the GM and host immunity have stabilized, the bacteria are less stressed and the temperate virome, in turn, less dominant. This notion is consistent with how a virulent phage core is linked to adult gut health^69^, as well as the paucity of crAssphage in infant viromes^29^.

## Methods

### The COPSAC2010 cohort

The study was embedded in the Danish population-based COPSAC2010 prospective mother–child cohort of 736 women and their children followed from week 24 of pregnancy, with the aim of studying the mechanisms underlying chronic inflammatory diseases^37^ (Supplementary Table 1). The study was conducted in accordance with the guiding principles of the Declaration of Helsinki and was approved by The National Committee on Health Research Ethics (H-B-2008-093) and the Danish Data Protection Agency (2015-41-3696). Both parents gave written informed consent before enrollment. Faecal samples were collected for 660 participants at age 1 year.

### Virome extraction

Each sample was mixed with 10% vol/vol glycerol and stored at −80 °C until DNA extraction for metagenomes^38^ and virome extraction. Extraction and sequencing of viromes were done using previously described protocols^70^. Briefly, DNA from faecal filtrates enriched in viral particles was extracted and subjected to short (30 min) MDA amplification and libraries were prepared following the manufacturer’s procedures for the Illumina Nextera XT kit (FC-131-1096). For epiflorescence virus-like particle (VLP) estimations, 10 µl of a virome sample was diluted 100-fold, fixed and deposited on a 0.02 µm filter, dried and stained with SYBR-Gold (200×), then visualized with an epifluorescence microscope using a 475 nm laser. VLPs were counted in eight to ten fields and multiplied over the remaining filter surface area.

### Sequencing, assembly and decontamination

Virome libraries were sequenced on the Illumina HiSeq X platform to an average depth of 3 Gb per sample with paired-end 2× 150 bp reads. Satisfactory sequencing results were obtained for 647 out of 660 samples. Virome reads were quality filtered and trimmed using Fastq Quality Trimmer/Filter v0.0.14 (options -Q 33 -t 13 -l 32 -p 90 -q 13), and residual Illumina adaptors were removed using cutadapt (v2.0). Trimmed reads were de-replicated using the VSEARCH^71^ (v2.4.3) derep_prefix and then assembled with Spades^72^ (v3.10.1) using the meta flag while disabling read error correction. Decontamination clusters were generated by reducing redundancy by de-duplicating the 1.5 M contigs above 1 kb in size into 267k 90% ANI representatives using a previously published pipeline^73^ then calling genes using Prodigal^74^ (v2.6.3) and aligning proteins all-against-all using FASTA^75^ (v36.3.6f) for building an APS tree^76^ using custom code (https://github.com/shiraz-shah/VFCs). The tree was cut close to the root to obtain the decontamination clusters. Bacterial MAGs from the same samples^38^ were mined for CRISPR spacers using CRISPRDetect^77^ (v2.2), and the virome decontamination clusters were ranked by their extent of CRISPR targeting multiplied by sample prevalence. The protein alignment results were passed through an orthology filter^78^ (https://github.com/shiraz-shah/VFCs) and clustered using Markov clustering^79^ (v14-137) to obtain VOGs de novo. VOGs were used to visualize the gene contents of contigs within each decontamination cluster. The top 400 ranked clusters were inspected visually for two viral signatures, namely conservation of contig sizes and of gene content. There were diminishing returns beyond the top 400 mark and the remaining decontamination clusters were assumed to represent contaminants.

### OTU delineation and protein annotation

Species-level (95% ANI) de-duplication of contigs into OTUs was done using BLAT^80^ and custom code for clustering (https://github.com/shiraz-shah/VFCs). Reference phages were de-duplicated to the species-level using the same strategy. Comparisons of the vOTUs to the GVD, GPD and MGV were also performed similarly. Decontaminated vOTUs and reference phage species^41^ were pooled and the APS tree and VOGs were recomputed. Multiple sequence alignments (MSAs) of VOGs were constructed with MUSCLE^81^ v3.8.425. VOG MSAs were aligned against MSAs from Pfam^82^, the Conserved Domains Database^83^, the Clusters of Orthologous Groups of proteins database^84^ and TIGRFAMs^85^ using HH-suite3 (ref. ^86^) v3.0-beta.3 to gain functional annotations.

### Resolution of viral taxonomy

We first used FigTree (v1.4.4) to root the APS tree by selecting an outgroup that branched out directly from the stem of the tree. Next we used phylotreelib and treetool (https://github.com/agormp/phylotreelib) to generate viral genera, subfamilies, VFCs and VOCs as follows. First, treetool’s cladeinfo option was used to retrieve the distances from the root to the branch points corresponding to existing phage genera, subfamilies, families and orders^32,35^. Next, treetool.py’s–clustcut option was used to cut the rooted APS tree at the above distances in order to obtain clades of both vOTUs and reference phages corresponding to viral genera, subfamilies, families and orders. The distances we used to cut the tree were 0.250, 0.125, 0.04 and 0.025, respectively, corresponding to average amino-acid identity (AAI) and coverage thresholds of 70%, 50%, 28% and 22% for each respective taxonomic level.

### Curation of VFCs

Viral families from above were visualized (Extended Data Fig. 2) to (1) further curate each individual member vOTU to separate confirmable viruses that had structural VOGs, from subclades of vOTUs representing various virus-related MGEs, such as satellites, that did not harbour genes coding for typical structural proteins. (2) The OTU length distribution within each family was inspected and then plotted in a histogram with 5 kb steps to locate the right-most size peak. The 5 kb step immediately preceding this peak was set as the lower size bound for a complete or near-complete genome. (3) The family visualizations were inspected to manually remove families that were dominated by reference phages, so as to avoid interference with ongoing classification efforts. Weak families composed mainly of MGEs or fragments, having fewer than five vOTUs or fewer than two complete vOTUs were also removed. For the final version of the family visualizations available online, VOG MSAs were realigned against MSAs from PHROGs^87^ because this database was more informative than Pfam, Conserved Domains Database, Clusters of Orthologous Groups of proteins database and TIGRAMs.

### Host prediction

MAG spacers, along with spacers from CRISPRopenDB^57^ and WIsH^57^ (v1.0) were used to generate separate host predictions for each vOTU. The three predictions were integrated using the last common ancestor of the two most closely matching predictions, as an error-correction strategy, since all three methods would occasionally mispredict. Bacterial genus abundances in the metagenome were derived by running mOTUs^88^ (v2) on the reads from each sample followed by aggregating mOTU abundances at the genus-level in R (v4.0.2) using phyloseq^89^ (v1.41.1).

### Abundance estimation

Bacterial contamination was estimated for each virome sample using ViromeQC^40^ (v1.0) along with a custom approach where we leveraged the metagenomes cognate to each virome: Reads were mapped from both fractions against the 16S rRNA gene^90^ and *cpn60* (ref. ^91^) and the degree of contamination was calculated as the ratio between the two fractions. Abundances of vOTUs in each sample were determined by mapping sample reads to sample contigs using the Burrows–Wheeler Aligner^92^ (v0.7.17-r1188) with the option mem -a, then using the msamtools (v0.9.6) profile to determine depth and length-normalized relative abundances with iterative redistribution of ambiguously mapped reads proportionally to uniquely mapped reads (https://github.com/arumugamlab/msamtools). The obtained contig abundances were then aggregated at the OTU level using custom code (https://github.com/shiraz-shah/VFCs) to obtain vOTU abundances per sample. vOTU abundances were aggregated at the family and order levels in R (v4.0.2) using phyloseq^89^ (v1.41.1) to obtain the statistics used for Figs. 2 and 3.

### Phage lifestyle prediction

A list of VOGs matching to integrase and large serine recombinase protein families was first curated, then used to predict whether complete vOTUs within viral families were temperate or virulent. Families where more than 95% of complete vOTUs did not harbour an integrase were deemed virulent, whereas for temperate families at least 50% of both complete and incomplete vOTUs were required to carry an integrase.

### Benchmarking

The versions of virus discovery tools used for benchmarking (Supplementary Table 2) were DeepVirFinder (v1.0), VIBRANT (v1.2.1), VIRSorter (1.0.6), VIRSorter2 (v2.0 commit 22f6a7d), Seeker (commit 9ae1488), PPR-Meta (v1.1) and CheckV (v.0.7.0). The random prediction was created by randomly sampling the 362,668 OTUs 12,500 times without replacement. The number 12,500 was chosen because it was reasonably close to our own positive set and the number of positives generated by most tools.

### Figures and statistical analysis

Figure 1 was drawn by first collating data at the family level using phyloseq^89^ then using Circos v0.69-8 (ref. ^93^) for rendering. Figures 2–4, Extended Data Figs. 4–8 and corresponding statistical analyses were generated using the statistical software R and the tidyverse suite, including ggplot2 (ref. ^94^) and related add-on packages ggraph^95^, ggforce^96^, ggpubr^97^, ggrepel^98^, ggstance^99^ and patchwork^100^. For deriving unique branch lengths (Fig. 4), we used the function pd.calc from the caper package^101^. The neutral.fit function from the MicEco R library (https://github.com/Russel88/MicEco) was used for fitting the family-level abundances to the neutral community model.

### Availability of unique biological materials

Access upon request of the infant faecal samples to third parties is not part of the consent granted by the parents upon enrollment into the COPSAC2010 cohort. Nor is such access compliant with Danish or EU regulations for safeguarding rights of underage human research participants. Materials might however be obtained as part of a scientific collaboration agreement with COPSAC, and queries for such may be sent to the COPSAC Data Protection Officer, Ulrik Ralkiaer, PhD (administration@dbac.dk).

### Reporting summary

Further information on research design is available in the Nature Portfolio Reporting Summary linked to this article.

## Supplementary information

Supplementary Information

Reporting Summary

## Data Availability

Viral genome sequences, taxonomy and host predictions and VOGs for all viruses are available through the online version of Fig. 1 on http://copsac.com/earlyvir/f1y/fig1.svg as well as the FigShare repository 10.6084/m9.figshare.21102805. Benchmarking data including the non-viral sequence clusters is also available through the above as well as via http://copsac.com/earlyvir/f1y/benchmark.tsv. Sequencing FASTQ files can be accessed through the European Nucleotide Archive (ebi.ac.uk) using the project number PRJEB46943. Reference phages were obtained from the INPHARED database on millardlab.org. Reference Bacterial *cpn60* sequences were obtained from cpndb.ca.

## Code availability

Data analyses were carried out using free and open source software as specified in the Online Methods. Custom code was also used and is available on GitHub (https://github.com/shiraz-shah/VFCs).
