# Supplementary material for: Expanding known viral diversity in the healthy infant gut
Source: Nat Microbiol. 2023 Apr 10;8(5):986–98. doi: 10.1038/s41564-023-01345-7 (PMC10159846; doi:10.1038/s41564-023-01345-7)
Supplement: Supplementary file 1 — Supplementary Information [file 41564_2023_1345_MOESM1_ESM.pdf]

nature microbiology

Resource

https://doi.org/10.1038/s41564-023-01345-7

Expanding known viral diversity in the healthy infant gut

In the format provided by the authors and unedited

Supplementary information

Tables

Table S1: Baseline characteristics of the children included in the analyses.

**characteristic**	**n (%)**
total	647
Male (%)	337 (52.1)
Ethnicity (Northern European) (%)	617 (95.4)
Mother's age (yr, mean (SD))	32.17 (4.30)
Household income (%)	
Low	62 (9.6)
Medium	343 (53.1)
High	241 (37.3)
Atopy mother (%)	343 (53.1)
Pregnancy antibiotics (%)	235 (36.4)
Delivery type (%)	
Vaginal	510 (78.8)
Emergency section	78 (12.1)
Elective section	59 (9.1)
Gestational age (weeks, mean (SD))	39.86 (1.68)
Birth length (cm, mean (SD))	51.89 (2.54)
Birth weight (kg, mean (SD))	3.54 (0.55)
Birth season (%)	
autumn	139 (21.5)
spring	170 (26.3)
summer	136 (21.0)
winter	202 (31.2)
Urban living environment (%)	342 (52.9)
Antibiotics during first year (%)	297 (46.3)
Breastfeeding total (days, mean (SD))	249.09 (165.45)
Breastfeeding exclusive (days, mean (SD))	104.16 (59.40)
Age at virome sample (yr, mean (SD))	1.02 (0.09)

Table S2: Benchmarking statistics from various metagenomic virus discovery methods against 10,021 vOTUs in the manually curated viral set from a total of 362,668 95% ANI sequence clusters. The imbalanced nature of the data set, with non-viral sequence clusters outnumbering vOTUs, inflates the specificity metric, which is 0.965 for a random prediction. The Kappa performance metric was included because it is robust against such imbalances. Prediction methods that provided a confidence score were cut according to authors' recommendations where available, or else by matching the number of positive predictions to the manual set. The performances of VIRSorter and VIBRANT were also checked in alternate modes (bottom portion). The raw data for the benchmark can be obtained from http://copsac.com/earlyvir/f1y/benchmark.tsv.

method	predictions	cutoff	sensitivity	specificity	kappa
CheckV	9393	>= medium qual	0.71161	0.99359	0.7273
viralVerify	10921	> 15	0.69873	0.98889	0.6589
VIBRANT	5843	>= medium qual	0.44696	0.99613	0.5556
VIRSorter	14366	cat. 1 + 2	0.69335	0.97896	0.5553
Length + 20kb	13452	> 20kb	0.48548	0.97565	0.3954
VIRSorter2	18638	virus maxscore = 1	0.50983	0.96164	0.3325
DeepVirFinder	10192	q < 0.05	0.058078	0.972749	0.0306
PPR Meta	17222	score > 0.95	0.060772	0.952891	0.0101
random	12500	w/o replacement	0.0330306	0.9654924	-0.0013
Seeker	13520	>= 0.9	0.0263447	0.9624100	-0.0096
VIRSorter virome	30736	cat. 1 + 2	0.83155	0.93647	0.3832
VIRSorter db2	13298	cat. 1 + 2	0.70971	0.98246	0.5973
VIBRANT virome	6181	>= medium qual	0.45395	0.99537	0.5521

Table S3: The 10,021 viral species belonging to the 248 viral families were grouped into viral order-level clades (VOCs). VOCs are sorted here by total richness. Caudoviral mean read abundances (cMRA) are shown for the caudoviral VOCs. For each VOC the number of viral family clades (VFCs), subfamilies, and genera are also given along with their most frequent hosts.

**order-level clade**	**% cMRA**	**VFCs**	**subfamilies**	**genera**	**vOTUs**	**major host**
VOC1	27	67	531	1,163	2,719	*Clostridiales*
Petitvirales	-	7	67	154	1,424	*Bacteroides*
VOC2	19	45	315	618	1,417	*Clostridiales*
VOC3	9	30	171	358	1,031	*Veillonellaceae*
anelloviruses	-	1	21	268	970	human
Crassvirales	15	30	114	214	561	*Bacteroidales*
VOC4	7	17	64	149	502	*Bifidobacterium*
VOC5	6	8	47	108	334	*Bacteroidales*
VOC6	5	4	27	75	278	*Clostridiales*
Tubulavirales	-	7	50	78	235	*Clostridiales*
VOC7	2	11	37	61	145	*Clostridiales*
VOC8	1	10	38	61	111	*Clostridiales*
VOC9	1	7	19	31	96	*Akkermansia*
VOC10	6	1	4	10	93	*Bacteroides*
VOC11	1	1	3	4	92	*Veillonellaceae*
VOC12	0.05	1	3	6	8	*Faecalibacterium*
Rowavirales	-	1	1	2	5	human

```
 initn:  42 init1:  42 opt:  42  Z-score: 66.6  bits: 21.5 E(2): 0.13
Smith-Waterman score: 60; 24.8% identity (51.3% similar) in 113 aa overlap (215-323:226-331)

                       190       200       210       220        230       240
OTU_3060_5          KHWEWFTNTMLSRLEENGKIIIMTRWASDDLAGRAIAHY-TQQGVNVRHITMKALIDKE
                                                  ::  : :    : :: . ....  : .:
OTU_3175_18         QRKMLAAPKGTKLYKNKILGLRGRATGLVFDLQPRNIISLGTAQGFKFERFS--AGLDTA
                      200       210       220       230       240        250

                        250         260       270       280       290        300
OTU_3060_5          KHTMLCPEIL--SYVSYLAKTKAMGVDIASANYQQEPIDLKGRLYDSFKTYTE-LPKDSN
                     ...  :. .  ..:.  :  : . .:  . : ... : :      :  : .:: : :.
OTU_3175_18         -YSQSSPDTIAFTFVGITADRKCVTLDEETYNNRDRQIPLTPS--DIPKIFTEFLEKNRR
                         260       270       280       290         300       310

                          310       320       330       340       350       360
OTU_3060_5          GNSLFEGIYSYTDTADEGDDFLCSIIWGVYMREAYVLDVYFSKANMEITEKETARRHKEF
                      .. .  .:   :.::..  . :
OTU_3175_18         LWGFAKDVY--IDSADQATILECQKFKRLSGSLYNFIPAFKKTKIIDRIHLQSAWLAAGD
                             320       330       340       350       360
```

```
initn: 2503 init1: 1900 opt: 2506  Z-score: 1933.2  bits: 367.2 E(2): 1.4e-105
Smith-Waterman score: 2506; 76.9% identity (90.4% similar) in 490 aa overlap (3-492:5-491)

                               10        20        30        40        50
OTU_3060_4            MKIGERFKSMIQNWLNIVPAANQSVVLQELLPREIEVLRSQLWYRGDATELRQFFHQI
                        :::. ..:. ::::: ::..:. ::.:      ..::..::::::: :: :::.:.
OTU_3175_17         MKFDIGEKVRQMFLNWLNINPASEQTFVLNERTGLMADILRAKLWYRGDAYELSQFFKQL
                            10        20        30        40        50        60

                    60        70        80        90       100       110
OTU_3060_4          GDGSGSFWASVPNKNNIRKIHSGLPAIIADTLAYIVYSDMDKIKVTGEKENSIFESVSKA
                    : :..:::.:::....::::::::::::::::::::::::: : : :::    ::..:.
OTU_3175_17         GCGTNSFWGSVPDNEKVRKIHSGLPAIIADTLAYIVYSDMDDIAVEGEKGRVAFEDISQN
                            70        80        90       100       110       120

                   120       130       140       150       160       170
OTU_3060_4          VDFNELVGKAVVDTLVSGDGAFKISVDMTENSVSDVPIVEFWSADKVEYRYIRGVLKEVV
                    .::. :::::.::::: ::::::::::   ...: .::::: .:::.::::.::::.::.
OTU_3175_17         TDFTALVGKAIVDTLVEGDGAFKISVD---DTLSLTPIVEFVGADKIEYRYLRGVLSEVI
                           130       140          150       160       170

                   180       190       200       210       220       230
OTU_3060_4          FRSEHKEGDRLYHLEECYGKGYIESRLYDNSGHEVRLDSVPCLSGIETRTIFDGDYIMAV
                    ::: :..:.:.:.::: ::.:::::::::.::::: ::::::::.::: :. :  ::::::
OTU_3175_17         FRSAHEDGNRIYQLEEHYGRGYIESRLYDHSGHEVSLDSVPCLAGIEPRVEFARDYIMAV
                    180       190       200       210       220       230

                   240       250       260       270       280       290
OTU_3060_4          PLKFYASKKYPNRGKSIFDGGKSDCFDALDEVISQWWDAIRAGRVTKYIPSDKIPRSPEN
                    ::::::::::.:::::::::::::::::::::::::::::::: ::: .:::.. :::. ::
OTU_3175_17         PLKFYASKKYPGRGKSIFDGGKSDCFDALDEVISQWWDAIRMGRVKQYIPDNMIPRNAEN
                    240       250       260       270       280       290

                   300       310       320       330       340       350
OTU_3060_4          GALQRVNSFGNEFIEIASSLGDERSSQIQVVQPDIKYDAFVSSYTNCLLMCLQGLVSPAT
                    :.. ..:.:::..: :.. : .  . .:.::::::::::::::::::::::::::::::::
OTU_3175_17         GSVGKLNQFGNNYITISQPLQEGVTPKIEVVQPDIKYDAFVSSYTNCLLMCLQGLVSPAT
                    300       310       320       330       340       350

                   360       370       380       390       400       410
OTU_3060_4          LGIDVGKMSSADAQREKKDVTGNTRNTITTALEKALPELVSAVLKTYDNMQGKAPEEYEV
                    :::::::.::::::::::::::::::::::::::::::::::::::::::::::::::::
OTU_3175_17         LGIDVGKMQSADAQREKKDVTGNTRNTITTALEKALPELVSAVLKTYDNMQGKAPEEYEV
                    360       370       380       390       400       410

                   420       430       440       450       460       470
OTU_3060_4          SVDFGEYGAPDFDSRVETVGKASTYGIMSVETQVEELWGSSKEDEWKAGEVKRIMQEKGL
                    ::::::::::::::::::::::::::::::::::::::::::::::::::::::::::::
OTU_3175_17         SVDFGEYGAPDFDSRVETVGKASTYGIMSVETQVEELWGSSKEDEWKAGEVKRIMQEKGL
                    420       430       440       450       460       470

                   480       490
OTU_3060_4          ADGTTPAVGDELA*
                    :::.: ::::::::
OTU_3175_17         ADGATSAVGDELA*
                    480       490
```

Figure S1: Alignments of TerL and the portal protein for two viruses belonging to the same genus within the *Jeppeviridae* family. The 75% amino acid identity for the portal protein alignment is within the expected range for phages of the same genus, whereas the terminase proteins are so disparate that gene exchange is the most likely explanation.

Supplementary Results

Viral densities in the infant gut

In order to gauge the quality of the virome extractions we estimated the virus particle concentration for a subset of the samples using epifluorescence microscopy. The mean virus-like particle (VLP) concentration obtained was $1.0 \cdot 10^9$ VLPs/g of faeces , ranging from $3 \cdot 10^8$ to $3 \cdot 10^9$ VLPs/g for the 18 samples tested.

The gut microbiome is shaped through infancy and protects against chronic disease later in life. Bacterial viruses (phages) modulate bacterial growth by lysis and lysogeny. Viral metagenomes (viromes) are still difficult to analyse because they span uncharted viral diversity. Here, we systematically resolved the uncharted viral diversity in faecal samples from 647 one-year-olds. Their deeply sequenced viromes were characterised through assembly and curation, uncovering thousands of viral species in 248 virus family-level clades (VFCs). The majority (232) of these were previously unknown and belonged to the *Caudoviricetes* viral class. Hosts were determined for 79% of the phages using CRISPR spacers within bacterial metagenomes from the same children. Typical *Bacteroides*-infecting crAssphages were outnumbered by novel viral families including phages infecting *Clostridiales* and *Bifidobacterium*. Phage lifestyles were largely conserved at the virus family-level with 33 families being virulent, while 118 were temperate. Virulent phages were more abundant, while temperate ones were more prevalent and diverse. Together, the viral families found in this study represent a useful expansion of existing bacteriophage taxonomy aiding future translational infant gut viromics research.

Detection of ssDNA viruses while preserving quantitative abundances

Before library preparation we used multiple-displacement amplification (MDA) to capture ssDNA viruses. MDA is believed to introduce compositional biases[1] and compromise quantitative analyses[2]. To control these effects we shortened the MDA step (sMDA) down to 30 minutes instead of the recommended two hours. After sequencing and assembly, vOTU abundances were estimated by read mapping and normalising for mapping depth and contig length. To investigate whether the sMDA had compromised caudoviral abundances, we compared counts of plaque forming units (PFUs) for 15 virulent coliphages[3] against mapped reads from both the sMDA amplified virome and non-amplified metagenome. Both data sets were quantitative over the entire range of PFU measurements (250 to 1M PFU/g) (Figure S9). Thus, the sMDA protocol did not compromise quantitative analysis of the dsDNA virome, allowing for valid comparisons of viral abundances across different vOTUs and samples.

Manual decontamination and OTU delineation

Assembly of all samples yielded 1.5M contigs in total and the contigs were first dereplicated at 90% nucleotide identity and coverage, resulting in 267k OTUs. Protein coding genes from all OTUs were compared in an all-against-all alignment search for two purposes: First VOGs

were delineated *de novo* and annotated with profile-profile alignments against major protein family databases. Secondly, alignments were used to cluster the OTUs by remote protein similarity, yielding 8050 deep "decontamination clusters".

Decontamination clusters were inspected manually. To avoid manual inspection of all 8050 clusters they were ranked based on aggregate prevalence across samples and extent of CRISPR targeting from the metagenome. Using the VOGs, we made genome maps of all OTUs in each cluster (similar to Extended data figure 2). We inspected the 400 top-ranking clusters manually, and found 255 of them to appear viral (criteria below). Diminishing returns beyond the top 400 mark meant that no additional decontamination clusters were inspected. Of the clusters we did inspect and that were not viral, 64 appeared to be plasmid clusters (e.g. encoding partitioning proteins and type IV secretion systems), while the remainder appeared spurious and were assumed to comprise bacterial contamination. Viral clusters encoded viral structural proteins and were conserved in genome size and gene content and plasmid clusters were much more heterogeneous while contaminant clusters were distinctly short. The 255 viral decontamination clusters comprised 8296 vOTUs at the 90% ANI-level, or 16,746 species-level (95% ANI) vOTUs.

After viral family-level clustering of the 16,746 vOTUs, we found that some VFCs were composed of both viruses and various MGEs of viral origin. The latter appeared to be either satellite phages or other MGEs employing viral polymerases making them co-cluster within the viral VFCs. Thus all vOTUs were curated individually to remove such viral MGEs (criteria in Additional Methods). Further, VFCs corresponding to known viruses (e.g. Lamba-like phages, Tevenvirinae, etc.) were deliberately discarded in order to avoid interference with ongoing virus classification efforts. In the end the final set of vOTUs was reduced to 10,021 manually confirmed viral species distributed over 248 VFCs.

Estimating the ratio of bacterial contamination

The 10,021 confirmed vOTUs recruited roughly half of the reads despite making up a minor fraction of all OTUs (Extended data figure 3). The remaining half of the read mappings were spread over the 346k non-viral sequence clusters. Since ViromeQC[4] estimated a mean bacterial contamination rate of 44% at the read-level, we infer that most non-viral sequence clusters must be from bacterial contamination (Extended data figure 3). After subtracting viral and bacterial reads, only 7% of the reads were left unaccounted.

Phage-host abundances are linked both within samples and study-wide

In order to determine whether the abundance of phages infecting a particular host was associated with the abundance of the host itself, we correlated phages and hosts at both the sample-level and study-wide. First individual vOTU relative abundances were aggregated by predicted host genus. These were then compared to bacterial genus abundances in metagenomes for the corresponding samples using Spearman tests (Extended data figure 7). Correlations between corresponding phage-host genus pairs were enriched for positive values across all genera (Extended data figure 7A, $p = 2.4 \cdot 10^{-12}$), indicating positive co-abundance for phages and their hosts between children. Positive associations between

phage-host pairs were expected for temperate phages actively induced by their hosts, and since such phages were more common in our data (4800 temperate vs. 1006 virulent vOTUs), they could be masking any negative signal from virulent phages killing their hosts. After splitting up the vOTU data by predicted phage lifestyle and recomputing correlations between pairs of phage-host genera, both lifestyles still yielded strong positive correlations (Extended data figure 7C).

Next, study-wide mean relative abundances (MRAs) were calculated by averaging the relative abundances for each vOTU across all samples. These were also aggregated at the host-genus level and compared with MRAs for bacterial genera in the metagenomes, yielding a strong positive correlation (Spearman's $\rho = 0.76$, $p < 2.2 \cdot 10^{-16}$, Figure 4C). A similar result was obtained even after splitting the data into temperate and virulent phages (Extended data figure 8).

Benchmarks of virome decontamination software

A series of virus discovery and decontamination tools have been published recently[4–12] and they have already seen widespread applications. Although most of the tools are meant for use on metagenome assemblies, or predicted (pro-)phage genomes, they are often applied directly to virome assemblies for decontamination purposes.

Several large gut phage databases have been built on predictions from such tools[13,14]. Yet, little is known about their efficacy in identifying novel viral clades or their ability to weed out contaminating DNA. The manually curated nature of our virome data set made it well suited for independently testing the performance of these tools. A purely random prediction was generated for comparison. A naive length cutoff of +20 kb was also used for comparison, since non-viral contaminant sequence clusters were distinctly short in our data (Extended data figure 4).

CheckV[6], VIBRANT[5] and viralVerify[8] sported the best performances with our data set (Table S2) although VIRSorter[11] also worked well. With a specificity of 97.5%, the length cutoff did a better job than VIRSorter2[7]. DeepVirFinder[12] PPR-Meta[10] and Seeker[9], all of which were "alignment free", yielded performances that were close to random. VIRSorter and VIBRANT, when run in virome decontamination mode, improved sensitivity at the cost of greatly reduced specificity. For our data, VIRSorter performed better when used in "db2" mode.

ViromeQC[4] estimated a mean bacterial contamination rate of 44% at the read-level (Extended data figure 3). The median [IQR] contamination rate reported by ViromeQC was 36% [22% - 54%] and this range is similar to a test of published viromes by the authors of ViromeQC. We compared the ViromeQC estimation to two other independent measures generated using the manual curation and our coupled metagenome samples, namely 1) the proportion of mapped reads to non-viral contaminant sequences clusters (median [IQR] 42% [24% - 64%]) and 2) the depletion of bacterial core genes in the virome reads compared to cognate metagenomes (median [IQR] 37% [19% - 46%]). Although all three estimates were in strong overall agreement, there was considerable sample to sample variation (Extended data figure 5). Importantly, our metagenome-normalised core gene depletion did not perform better than ViromeQC when comparing against the manually validated non-viral mapping. This result illustrates that virome contaminant estimation is non-trivial even when bulk

metagenomes are available for the same samples, and that ViromeQC performs well considering all this uncertainty.

Supplementary discussion

Bacterial contaminating DNA made up around half of our sequenced virome reads, which is within the typical range[4]. After assembly and species-level deduplication, the total number of non-viral sequence clusters was 20 times greater than our total number of viral OTUs (Extended data figure 3). vOTUs were longer and more prevalent than contaminating bacterial sequence clusters which tended to be sample-specific (Extended data figure 4). Random segments of bacterial chromosomal DNA likely became copurified along with the viral particles, explaining why they were not generally conserved between samples. Contaminant DNA species thus made up the majority of the overall sequence diversity but were shorter and much less prevalent than the viruses. Setting cutoffs on OTU length and prevalence may thus provide an effective means for virome decontamination in future studies.

For resolving the taxonomy of our vOTUs into genera, subfamilies, families and order-level clades we used an amino acid identity (AAI) based phylogenomic approach and applied global cutoffs after rooting. Although it has been argued that global cutoffs are not suitable for virus classification[15] we found they worked well, and they came with the key advantage of reproducibility at multiple taxon levels. A different phylogenomic approach based on concatenated protein phylogeny was also remarkably successful at various taxonomic ranks among *Caudoviricetes*[16]. An alternative method for automatic genomic classification relies on gene-sharing networks rather than trees, and provides satisfactory results at the genus level[17], while at the family level, manual inspection of the network is required[18]. The existing guideline for defining new phage families[18] involves manual inspection of gene-sharing networks[17], and a reproducible alternative would be preferred. A common concern about clustering viruses based on shared proteins is the co-clustering of phages sharing accessory rather than core genes. However, to this day phylogenomics has proven a robust method for phage classification as it resists the formidable capacity of phages to exchange genetic material.

Although the large terminase subunit (TerL) was the most conserved protein in our caudoviruses, its gene was frequently exchanged such that even members of the same viral genus would carry different TerL homologs (Extended data figure 10). Notable examples of this phenomenon are found in VOC1. Thus, the practice[19,20] of using TerL phylogeny to classify caudoviral phages can sometimes produce confusing results. As shown by Yutin *et al.*[20], *Crassvirales* is not TerL monophyletic and non-Crass phages often encode TerLs that wind up in the middle of the crAss TerL tree. The recent introduction of *ε-crassviridae* into *Crassvirales*[20] may illustrate this problem, as our results suggest that the family may not be a crAssphage family (Figure 1).

Finally, we found that the latest generation of metagenome virus discovery tools such as CheckV, viralVerify and VIBRANT, in conjunction with ViromeQC should account for most sequences in one's virome data (Table S2), rendering any "virome dark matter" almost non-existent, at least for our data. Although the sensitivities of the tools against our data never got close to 100%, most of the sequences missed by the best tools were simply too short to pass the imposed quality thresholds. Thus, their predictions were good and certainly easier to obtain than manual curation. On the other hand, the performances of the

alignment-free methods were very close to random with our dataset, and it appears that nucleotide-level motifs do not carry the information required to reliably distinguish viruses from their abundant hosts.

References

1. Roux, S., Emerson, J. B., Eloe-Fadrosh, E. A. & Sullivan, M. B. Benchmarking viromics: an evaluation of metagenome-enabled estimates of viral community composition and diversity. *PeerJ* **5**, e3817 (2017).
2. Yilmaz, S., Allgaier, M. & Hugenholtz, P. Multiple displacement amplification compromises quantitative analysis of metagenomes. *Nat. Methods* **7**, 943–944 (2010).
3. Mathieu, A. *et al.* Virulent coliphages in 1-year-old children fecal samples are fewer, but more infectious than temperate coliphages. *Nature Communications* vol. 11 Preprint at https://doi.org/10.1038/s41467-019-14042-z (2020).
4. Zolfo, M. *et al.* Detecting contamination in viromes using ViromeQC. *Nat. Biotechnol.* **37**, 1408–1412 (2019).
5. Kieft, K., Zhou, Z. & Anantharaman, K. VIBRANT: automated recovery, annotation and curation of microbial viruses, and evaluation of viral community function from genomic sequences. *Microbiome* **8**, 90 (2020).
6. Nayfach, S. *et al.* CheckV assesses the quality and completeness of metagenome-assembled viral genomes. *Nat. Biotechnol.* (2020) doi:10.1038/s41587-020-00774-7.
7. Guo, J. *et al.* VirSorter2: a multi-classifier, expert-guided approach to detect diverse DNA and RNA viruses. *Microbiome* **9**, 37 (2021).
8. Antipov, D., Raiko, M., Lapidus, A. & Pevzner, P. A. Metaviral SPAdes: assembly of viruses from metagenomic data. *Bioinformatics* **36**, 4126–4129 (2020).
9. Auslander, N., Gussow, A. B., Benler, S., Wolf, Y. I. & Koonin, E. V. Seeker: alignment-free identification of bacteriophage genomes by deep learning. *Nucleic Acids Res.* **48**, e121 (2020).
10. Fang, Z. *et al.* PPR-Meta: a tool for identifying phages and plasmids from metagenomic fragments using deep learning. *Gigascience* **8**, (2019).
11. Roux, S., Enault, F., Hurwitz, B. L. & Sullivan, M. B. VirSorter: mining viral signal from

microbial genomic data. *PeerJ* **3**, e985 (2015).

12. Ren, J. *et al.* Identifying viruses from metagenomic data using deep learning. *Quantitative Biology* vol. 8 64–77 Preprint at https://doi.org/10.1007/s40484-019-0187-4 (2020).
13. Gregory, A. C. *et al.* The Gut Virome Database Reveals Age-Dependent Patterns of Virome Diversity in the Human Gut. *Cell Host Microbe* **28**, 724–740.e8 (2020).
14. Camarillo-Guerrero, L. F., Almeida, A., Rangel-Pineros, G., Finn, R. D. & Lawley, T. D. Massive expansion of human gut bacteriophage diversity. *Cell* **184**, 1098–1109.e9 (2021).
15. Simmonds, P. *et al.* Consensus statement: Virus taxonomy in the age of metagenomics. *Nat. Rev. Microbiol.* **15**, 161–168 (2017).
16. Low, S. J., Džunková, M., Chaumeil, P.-A., Parks, D. H. & Hugenholtz, P. Evaluation of a concatenated protein phylogeny for classification of tailed double-stranded DNA viruses belonging to the order Caudovirales. *Nat Microbiol* **4**, 1306–1315 (2019).
17. Bin Jang, H. *et al.* Taxonomic assignment of uncultivated prokaryotic virus genomes is enabled by gene-sharing networks. *Nat. Biotechnol.* **37**, 632–639 (2019).
18. Barylski, J. *et al.* Analysis of Spounaviruses as a Case Study for the Overdue Reclassification of Tailed Phages. *Syst. Biol.* **69**, 110–123 (2020).
19. Benler, S. *et al.* Thousands of previously unknown phages discovered in whole-community human gut metagenomes. *Microbiome* **9**, 78 (2021).
20. Yutin, N. *et al.* Analysis of metagenome-assembled viral genomes from the human gut reveals diverse putative CrAss-like phages with unique genomic features. *Nat. Commun.* **12**, 1044 (2021).
