# Supplementary material for: Expanding known viral diversity in the healthy infant gut
Source: Nat Microbiol. 2023 Apr 10;8(5):986–98. doi: 10.1038/s41564-023-01345-7 (PMC10159846; doi:10.1038/s41564-023-01345-7)
Supplement: Supplementary file 2 — Reporting Summary [file 41564_2023_1345_MOESM2_ESM.pdf]

nature portfolio

Corresponding author(s): Dennis S. Nielsen

Last updated by author(s): Feb 8, 2023

Reporting Summary

Nature Portfolio wishes to improve the reproducibility of the work that we publish. This form provides structure for consistency and transparency in reporting. For further information on Nature Portfolio policies, see our Editorial Policies and the Editorial Policy Checklist.

Statistics

For all statistical analyses, confirm that the following items are present in the figure legend, table legend, main text, or Methods section.

n/a	Confirmed	
☐	☒	The exact sample size (*n*) for each experimental group/condition, given as a discrete number and unit of measurement
☒	☐	A statement on whether measurements were taken from distinct samples or whether the same sample was measured repeatedly
☐	☒	The statistical test(s) used AND whether they are one- or two-sided<br>*Only common tests should be described solely by name; describe more complex techniques in the Methods section.*
☒	☐	A description of all covariates tested
☒	☐	A description of any assumptions or corrections, such as tests of normality and adjustment for multiple comparisons
☐	☒	A full description of the statistical parameters including central tendency (e.g. means) or other basic estimates (e.g. regression coefficient) AND variation (e.g. standard deviation) or associated estimates of uncertainty (e.g. confidence intervals)
☐	☒	For null hypothesis testing, the test statistic (e.g. *F*, *t*, *r*) with confidence intervals, effect sizes, degrees of freedom and *P* value noted<br>*Give P values as exact values whenever suitable.*
☒	☐	For Bayesian analysis, information on the choice of priors and Markov chain Monte Carlo settings
☒	☐	For hierarchical and complex designs, identification of the appropriate level for tests and full reporting of outcomes
☐	☒	Estimates of effect sizes (e.g. Cohen's *d*, Pearson's *r*), indicating how they were calculated

Our web collection on statistics for biologists contains articles on many of the points above.

Software and code

Policy information about availability of computer code

Data collection	No software was used for data collection
Data analysis	fastx-toolkit 0.0.14, cutadapt 2.0, vsearch 2.4.3, SPAdes 3.10.1, blat v35, prodigal 2.6.3, FASTA 36.3.6f, mcl 14-137, MUSCLE v3.8.425, hhsuite v3.0-beta.3, rapidnj 2.3.0.2, bwa 0.7.17-r1188, samtools 1.9, msamtools 0.9.6, Circos v0.69-8, R 4.0.2 with libraries phyloseq, , ggplot2, ggraph, ggforce, ggpubr, ggrepel, ggstance, patchwork and custom code (https://github.com/shiraz-shah/VFCs)

For manuscripts utilizing custom algorithms or software that are central to the research but not yet described in published literature, software must be made available to editors and reviewers. We strongly encourage code deposition in a community repository (e.g. GitHub). See the Nature Portfolio guidelines for submitting code & software for further information.

Data

Policy information about availability of data

All manuscripts must include a data availability statement. This statement should provide the following information, where applicable:

- Accession codes, unique identifiers, or web links for publicly available datasets
- A description of any restrictions on data availability
- For clinical datasets or third party data, please ensure that the statement adheres to our policy

Viral genome sequences, taxonomy and host predictions and VOGs for all viruses are available through the online version of Figure 1 on http://copsac.com/earlyvir/f1y/fig1.svg as well as the FigShare repository https://doi.org/10.6084/m9.figshare.21102805. Benchmarking data including the non-viral sequence clusters is also

available through the above as well as via http://copsac.com/earlyvir/f1y/benchmark.tsv. Sequencing FASTQ files can be accessed through the European Nucleotide Archive (ebi.ac.uk) using the project number PRJEB46943. Reference phages were obtained from the INPHARED database on millardlab.org. Reference Bacterial cpn60 sequences were obtained from cpndb.ca.

Human research participants

Policy information about studies involving human research participants and Sex and Gender in Research.

Reporting on sex and gender	Although information on sex was collected for the participants of the cohort at birth in accordance with the ethics statement below, it was not used in this study. The aim of the current study was to explore the diversity of the human infant gut virome regardless of host sex.
Population characteristics	COPSAC2010 is a polulation-based mother-child cohort recruited in Copenhagen and Næstved, Denmark with the overall aim of studying the mechanisms that lead to chronic disease in childhood. The samples used here were faecal samples from 1-year-old infants.
Recruitment	The COPSAC2010 cohort is a population-based birth cohort of 700 children recruited in pregnancy and has been followed prospectively at the COPSAC research unit. Details on recruitment can be found in Bisgaard, H. et al. Deep phenotyping of the unselected COPSAC2010 birth cohort study. Clin. Exp. Allergy 43, 1384–1394 (2013) (which is also cited in the manuscript, reference 39)
Ethics oversight	The study was conducted in accordance with the guiding principles of the Declaration of Helsinki and was approved by The National Committee on Health Research Ethics (H-B-2008-093) and the Danish Data Protection Agency (2015-41-3696). Both parents gave written informed consent before enrollment.

Note that full information on the approval of the study protocol must also be provided in the manuscript.

Field-specific reporting

Please select the one below that is the best fit for your research. If you are not sure, read the appropriate sections before making your selection.

☒ Life sciences ☐ Behavioural & social sciences ☐ Ecological, evolutionary & environmental sciences

For a reference copy of the document with all sections, see nature.com/documents/nr-reporting-summary-flat.pdf

Life sciences study design

All studies must disclose on these points even when the disclosure is negative.

Sample size	The sample size was equal to the total size of the COPSAC 2010 cohort
Data exclusions	Infants for whom the delivered faecal samples were either exhausted after previous metagenomics analyses, or too small to perform a virome extraction were excluded from the study. After virome extraction and sequencing, samples that produced fewer than 50,000 reads were also excluded.
Replication	The criteria for the definition of viral taxa have been recently revised by the ICTV making replication difficult owing to the sparsity of independent studies that have switched over to the new criteria. However, out of the 248 found viral families, eight (Flandersviridae, Gratiaviridae, and alpha to zeta Crassviridae) were found recently in independent studies and thus replicate our family-definition criterion.
Randomization	Not relevant as the descriptive nature of the study means that we did not use any experiment and control groups.
Blinding	Blinding is not applicable to the current study as there was no group allocation. All cohort members were treated as a single group

Reporting for specific materials, systems and methods

We require information from authors about some types of materials, experimental systems and methods used in many studies. Here, indicate whether each material, system or method listed is relevant to your study. If you are not sure if a list item applies to your research, read the appropriate section before selecting a response.

Materials & experimental systems

n/a	Involved in the study
☒	☐ Antibodies
☒	☐ Eukaryotic cell lines
☒	☐ Palaeontology and archaeology
☒	☐ Animals and other organisms
☒	☐ Clinical data
☒	☐ Dual use research of concern

Methods

n/a	Involved in the study
☒	☐ ChIP-seq
☒	☐ Flow cytometry
☒	☐ MRI-based neuroimaging
